# The effect of acupuncture at the *Taiyang* acupoint on visual function and EEG microstates in myopia

**DOI:** 10.3389/fnint.2023.1234471

**Published:** 2023-11-15

**Authors:** Kangna Su, Lihan Wang, Zhongqing Wang, Jiayao Ma, Chao Zhang, Hongsheng Bi, Jianfeng Wu

**Affiliations:** ^1^Medical College of Optometry and Ophthalmology, Shandong University of Traditional Chinese Medicine, Jinan, China; ^2^Shandong Academy of Eye Disease Prevention and Therapy, Shandong Provincial Key Laboratory of Integrated Traditional Chinese and Western Medicine for Prevention and Therapy of Ocular Diseases, Shandong Provincial Clinical Research Center of Ophthalmology and Children Visual Impairment Prevention and Control, Shandong Engineering Technology Research Center of Visual Intelligence, Shandong Academy of Health and Myopia Prevention and Control of Children and Adolescents, Jinan, China; ^3^Ophthalmology Department of Northwest University First Hospital, Xi’an, Shaanxi, China; ^4^Affiliated Eye Hospital of Shandong University of Traditional Chinese Medicine, Jinan, China

**Keywords:** acupuncture, visual function, contrast sensitivity, microstates, EEG

## Abstract

**Objective:**

Acupuncture has certain effects to improve myopia visual function, but its neural mechanism is unclear. In this study, we acupunctured at the right *Taiyang* acupoint of myopic patients to analyze the effects of acupuncture on visual function and electroencephalographic activity and to investigate the correlation between improvements in visual function and changes in the brain.

**Methods:**

In this study, a total of 18 myopic patients were recruited. The contrast sensitivity (CS) of the subjects was examined before and after acupuncture, and electroencephalography (EEG) data of the entire acupuncture process were recorded.

**Results:**

The study found that compared with before acupuncture, the CS of both eyes in myopic patients at each spatial frequency was increased after acupuncture; compared with the resting state, the contribution of microstate C was decreased during the post-acupuncture state, and the transition probability between microstate A and microstate C was reduced; in addition, the contribution of microstate C was negatively correlated with CS at both 12 and 18 cpd.

**Conclusion:**

The contrast sensitivity of myopic patients was improved after acupuncture at the *Taiyang* acupoint (20 min), which may be related to microstate C.

## 1 Introduction

Acupuncture, as a supplementary alternative therapy, is often used for the treatment of eye disease. Studies have found that acupuncture can improve the mean light sensitivity of patients with non-arteritic anterior ischemic optic neuropathy and reduce the mean defect (Qin et al., 2015), as well as improve the visual acuity and contrast sensitivity (CS) of patients with retinitis pigmentosa and nystagmus (Bittner et al., 2014; Blechschmidt et al., 2017). Myopia is one of the common visual disorders that can lead to a loss of visual quality (Zorena et al., 2018), and the global prevalence of it has been increasing in recent years; it is estimated that the myopic population will account for 49.8% of the total global population by 2050 (Baird et al., 2020; Dong et al., 2020). Recent studies have shown that acupuncture at the *Taiyang* and other acupoints can improve the uncorrected distance visual acuity (UDVA) in myopia (Yachan et al., 2015; Zhuang et al., 2022; Table 1). Visual acuity reflects visual function only for high-contrast stimuli (Arikan and Kamis, 2022), whereas CS can also evaluate the visual function for low- and medium-contrast stimuli. Low and medium contrast visual functions are equally important in daily life (such as driving at night, reading newspapers, and identifying object boundaries, etc.) (Onal et al., 2008; Roark and Stringham, 2019; Zhuang et al., 2021); however, whether acupuncture can improve the CS of patients with myopia is still uncertain.

**Table 1:** The impact of acupuncture on visual function.

Study	Inclusion criteria	Acupoint	Results
Qin (2015)	Patients with non-arteritic anterior ischemic optic neuropathy	BL1 (Jingming), EX-HN7 (Qiuhou), GB14 (Yangbai), DU19 (Baihui), EX-HN1 (Sishencong), EX-HN5 (Taiyang), GB20 (Fengchi), LI4(Hegu), and SJ5 (Waiguan)	The mean light sensitivity was improved and the mean defect was reduced
Blechschmidt (2017)	Patients with retinitis pigmentosa	GV-20 (Bai hui), EX-HN3 (Yintang), CV-6 (Qihai), UB-18 (Ganshu), UB-20 (Pishu), UB-23 (Shenshu), GB-20 (Fengchi), LI-4 (Hegu), HT-7 (Shenmen), SI-3 (Houxi), LV-3 (Taichong), GB-34 (Yanglingquan), GB-37 (Guangming), SP-6 (Sanyinjiao), ST-36 (Zusanli), UB-1 (Jingming), ST-1(Chengqi), and Ex-HN7 (Qiuhou)	Visual acuity and CS were both improved
Bittner (2014)	Patients with nystagmus	GV-20 (Baihui), GV-24 (Shenting), EX-HN3 (Yintang), EX-HN5 (Taiyang), BL-2 (Cuanzhu), EX-HN4 (Yuyao), Ex-HN7 (Qiuhou), EX-HN14 (Yiming), ST-2 (Sibai), GB-20, ear (Eye1, Eye2, Eye, Liver), CV-12 (Zhongwan), CV-6 (Qihai), ST-25 (Taishu), LI-12 (Zhouliao), LI-3 (Sanjian), HT-8 (Shaofu) and BL-67 (Zhiyin)	Visual acuity and CS were both improved
Zhuang (2022)	Myopia	the corresponding auricular points of the heart, spleen, liver, kidney, and eye	UDVA was improved
Li (2015)	Myopia	auricular points: CO10 (kidney), LO5 (eye), TF4 (Shenmen), CO15 (heart), CO12 (liver), and CO13 (spleen)	UDVA was improved

A perceptual learning study has indicated that CS improvement was positively correlated with the training-induced contrast gain of neurons in striate cortex (V1) (Hua et al., 2010), and Spiegel et al. (2013) believed that anodal transcranial direct current stimulation (tDCS) in amblyopia can reduce activation in extrastriate cortex (V2 and V3) from the fellow eye input. First, the fellow eye means the control eye, which is the relatively good eye of the amblyopia (Spiegel et al., 2013), and selectively increases the cortical reflection of the amblyopic eye input, which in turn improves the contrast sensitivity of the amblyopic eye. Further research has shown that training-induced CS enhancement was negatively correlated with glutamine levels in visual areas (Jia et al., 2022). A study by Quentin et al. (2016) found that CS was improved after transcranial magnetic stimulation of the right frontal lobe. Ding and his colleagues confirmed that tDCS to higher-order brain areas (A7) can have a top-down effect, thereby enhancing the excitement of the V1 cortex, manifested as the improvement of CS (Ding et al., 2021). Therefore, acupuncture to improve the CS of myopic patients may not only be related to the visual area, but it may also involve broader brain regions or brain function networks.

Electroencephalography (EEG), as a non-invasive neuroimaging method, can study the dynamic changes of the brain in the milliseconds (Michel and Koenig, 2018; Li et al., 2022). EEG microstates refer to states in which the scalp potential field remains quasi-stable at a given moment or period (Koenig et al., 2002; Khanna et al., 2015; Michel and Koenig, 2018), and these states are closely related to the resting state networks of functional magnetic resonance imaging (fMRI) (Britz et al., 2010; Musso et al., 2010; Yuan et al., 2012; D’Croz-Baron et al., 2019). Studies based on fMRI show that acupuncture can regulate the activation state of the salience network (Huang et al., 2022; Lee and Chae, 2022) and alter the functional connectivity between different brain networks (Dhond et al., 2008; Ning et al., 2022). In addition, changes in microstates parameters (e.g., duration, contribution, and transition probability) induced by acupuncture can be used as objective indicators of acupuncture effects (Si et al., 2021), which can be used to reveal the modulatory effects of acupuncture on the nervous system of the brain (Zhang et al., 2023).

Studies have found that scalp acupoints are more likely to alter brain function compared with limb acupoints (Park et al., 2009) and, according to traditional Chinese medicine theory, the *Taiyang* acupoint (EX-HN5) is a common point for treating eye diseases and has the effect of brightening the eyes, relieving asthenopia, and promoting *qi* and blood circulation (Lam et al., 2011; Yang et al., 2012). Furthermore, studies (Bittner et al., 2014; Blechschmidt et al., 2017) have shown that acupuncture points such as the *Taiyang* improve visual functions like visual acuity and CS. In this study, we examined the UDVA and CS of myopic patients before and after acupuncture at the right *Taiyang* acupoint and used EEG to collect the subject’s brain electrical activity of the entire process of acupuncture, then observed the changes in the brain activation using EEG microstates methodology. In addition, the correlation between changes in microstates and visual function was analyzed.

## 2 Materials and methods

### 2.1 Subjects and recruitment

Inclusion criteria:

1. Aged 20 ~ 25 years old;
2. a spherical equivalent (SE) ≤ −0.50D, and > −6.00D (Note: SE = spherical+1/2cylinder);
3. cylinder power ≤ 1.50D and binocular anisometropia ≤1.00D;
4. subjects can accept wearing soft contact lenses to correct vision, and the best corrected visual acuity of the eye is 0.00logMAR or better;
5. except for myopia and astigmatism, there are no other lesions that affect vision;
6. right-handed.

Exclusion criteria:

1. Received acupuncture in the past 3 months;
2. subjects suffering from keratitis, conjunctivitis, glaucoma, nystagmus, small palpebral fissure, or combined with other ophthalmic organic diseases after ophthalmological examination;
3. suffering from attention deficit and hyperactivity disorder, claustrophobia, and neurological or psychiatric disorders;
4. the subject was unable to undergo the experimental protocol.

According to the inclusion and exclusion criteria, this study recruited 18 myopic patients, including nine males and nine females.

### 2.2 Visual function examination

The UDVA and CS of the subjects were examined before and after acupuncture (20 min), and the order of binocular examination was randomized.

#### 2.2.1 UDVA examination

UDVA was examined using a standard logarithmic visual acuity chart and then converted to logMAR visual acuity for recording.

#### 2.2.2 CS examination

The OPTEC 6500 functional acuity contrast test (Stereo Optical Co., Inc., Chicago, IL, USA) was used to examine CS. The equipment has five sets of different spatial frequencies (i.e., 1.5, 3, 6, 12, and 18 cpd), and each set of spatial frequencies consists of nine circular sine-wave gratings with contrast decreasing in 0.15 log units. Each grating contains three directions: vertical, a 15° tilt to the left, and a 15° tilt to the right, and the angle visual is 1.7°.

Without glare mode, choosing photopic conditions (85cd/m^2^), and setting a far vision (6m), and setting without glare mode. First the subjects wore soft contact lenses, and then a same examiner examined their monocular CS according to the low spatial frequency to high spatial frequency, high contrast to low contrast sequence, and the final recognized grating of each spatial frequency as its CS value (Arikan and Kamis, 2022).

### 2.3 Acupuncture method

**Figure 1:**
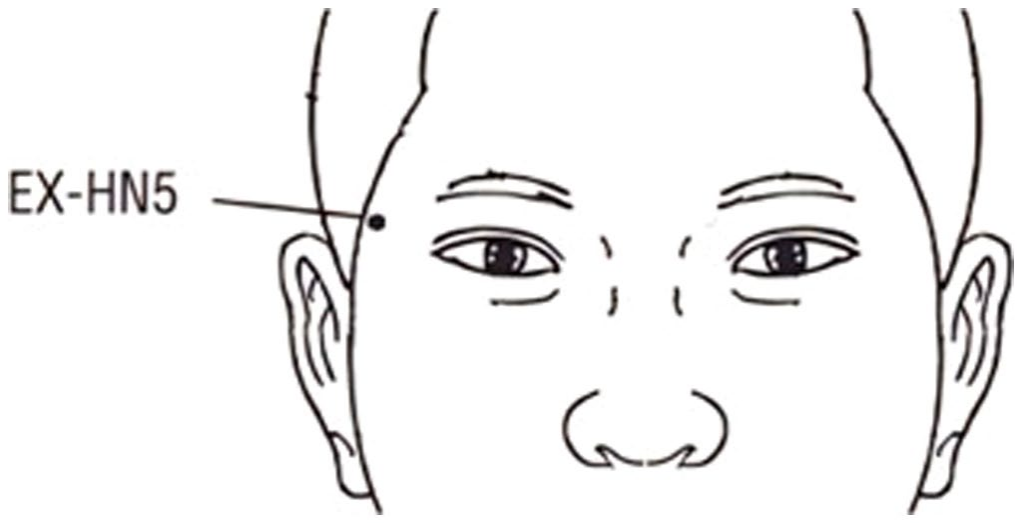
The location of the *Taiyang* acupoint.

**Figure 2:**
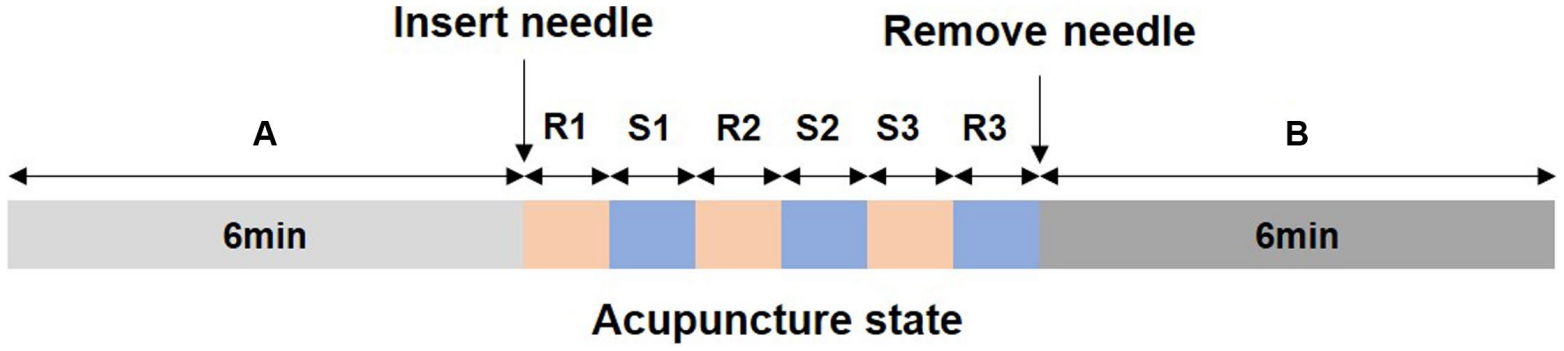
Acupuncture method; (A) and (B) represent the resting state and the post-acupuncture state, separately; (R1)–(R3) represents the needle inserted, and (S1)–(S3) represents stimulation. The time of both R and S is 1 min.

The acupuncture operation was performed by an experienced physician, using disposable sterile acupuncture needles (specification: 0.30 mm × 25 mm, Suzhou Huatuo Medical Co., Ltd.) to acupuncture the right *Taiyang* acupoint (at the temporal fossa, 1 *cun* behind the midpoint of a line from the lateral end of the eyebrow to the external canthus, Figure 1). The acupuncture method used retains the needle after inserting the needle to *deqi*, and then twisting manipulation is performed to achieve even reinforcing-reducing, with the frequency of twisting manipulation being 100 times/min. Both retention and stimulation time were 1 min, and the needle was removed after three repetitions (Chen et al., 2012). The entire acupuncture process was 18 min, including the resting state, acupuncture operation, and the post-acupuncture state, as shown in Figure 2.

### 2.4 EEG recording

The EEG signals were recorded by a NicoletOne with 29 channels in the international 10–20 system, and the reference electrode is Cpz. During the experiment, the impedance between the electrodes and the scalp was less than 5 kΩ, and the signal sampling rate was 1,024 Hz. The subjects sat quietly on a chair and were instructed to close their eyes, relax, and keep as static as possible during the collection process. EEG data were recorded during the entire acupuncture process, Figure 2.

### 2.5 Data analysis

#### 2.5.1 EEG data preprocessing

Data were extracted for 6 min from the resting state and the post-acupuncture state, respectively, then preprocessed using the EEGLAB (2021.1) toolbox in the MATLAB R2020a (The MathWorks, Natick, MA, United States) operating environment. First, a finite impulse response (FIR) filter was used for bandpass filtering in the 0.5–60 Hz range, followed by 50 Hz notch filtering to remove power line noise (Gao et al., 2018; Cui et al., 2021; Si et al., 2021; Zhao et al., 2022). Then, electromyography (EMG) and electrooculogram (EOG) were removed using blind source separation (BSS). Finally, the data were segmented into 2 s epochs, in which epochs with an amplitude of more than ±80 μV at any channel were rejected. As a result, the preprocessing procedures resulted in 176–180 (178 ± 2) 2-s epochs for the resting state and 176–180 (175 ± 14) 2-s epochs for the post-acupuncture state.

#### 2.5.2 EEG microstate analysis

The microstate analysis method considers the multichannel EEG electrodes as a series of quasistable microstates, and the topography of each state corresponds to the fMRI resting-state network. Currently, this method has been applied to the study of resting states (Gschwind et al., 2016; Chu et al., 2020; Xiong et al., 2021). EEG microstate analysis conducted with the Microstates 1.2 plug-in for EEGLAB. First, the last 4 min of both the resting state and the post-acupuncture state were chosen, and then the reference channels were converted to average reference (Koenig et al., 2002; Michel and Koenig, 2018). Second, to reduce artifacts in the EEG data, an FIR was used for further bandpass filtering from 2 to 20 Hz (Koenig et al., 2002). Then, the global field power (GFP) was calculated. GFP describes the standard deviation of all electrodes within a given time, and EEG data at the maximum value was selected as a discrete brain electrocardiogram microstate which has the best topographic diagram signal-to-noise ratio (equation 1; Si et al., 2021; Qiu et al., 2022). Thus, we only used data at the peak of the GFP for the follow-up analysis. Next, each subject data was analyzed separately by k-means clustering according to two conditions, the resting state and the post-acupuncture state (we set the re-calculating parameter to be 50), and to identify the specific group maps of different conditions, the individual cluster maps of all subjects were averaged by the permutation algorithm (Koenig et al., 2002; Kim et al., 2021). Referring to previous classic EEG microstate research, the number of microstate categories was set to four (Koenig et al., 1999, 2002; Hu et al., 2021), which can explain the most variance of the topographic map during the EEG microstate clustering. Then, we obtained the map topographies of different conditions. Finally, the parameters of the microstate (i.e., duration, occurrence, contribution, and transition probability) were calculated. Duration indicates that each microstate maintains a stable average time length when it appears; occurrence represents the number of times each microstate appears per second; contribution describes the proportion of the total time of each microstate; and transition probability indicates the probability of converting from one microstate to another (Croce et al., 2020; Ricci et al., 2022).

(1) $$GFP\left(t\right)=\sqrt{\frac{\Sigma_{i=1}^{N}v_{i}{\left(t\right)}^{2}}{N}}$$

(*N* = number of electrodes, *Vi(t)* = measured voltage of electrode *i* at time *t*, and *i* = electrode *i*).

### 2.6 Statistical analysis

Statistical analysis was conducted by SPSS version 26. For the UDVA, CS values, and microstate parameters, a separate two-sample *t*-test was conducted with false discovery rate (FDR) correction [*p_(FDR)_ < 0.05, **p_(FDR)_ < 0.01, ***p_(FDR)_ < 0.001]. Paired *t*-test was performed for transition probability between the resting state and the post-acupuncture state to check whether there were significant differences. Pearson’s correlation and linear regression analysis were conducted between the CS value of each spatial frequency and EEG microstate parameters.

## 3 Results

### 3.1 UDVA result

UDVA changes before and after acupuncture of both eyes had no statistical significance (Table 2).

**Table 2:** UDVA changes before and after acupuncture.

	OD	OS
Before acupuncture	0.657 ± 0.254	0.629 ± 0.272
After acupuncture	0.657 ± 0.256	0.605 ± 0.260

OD, right eye; OS, left eye.

### 3.2 CS result

**Figure 3:**
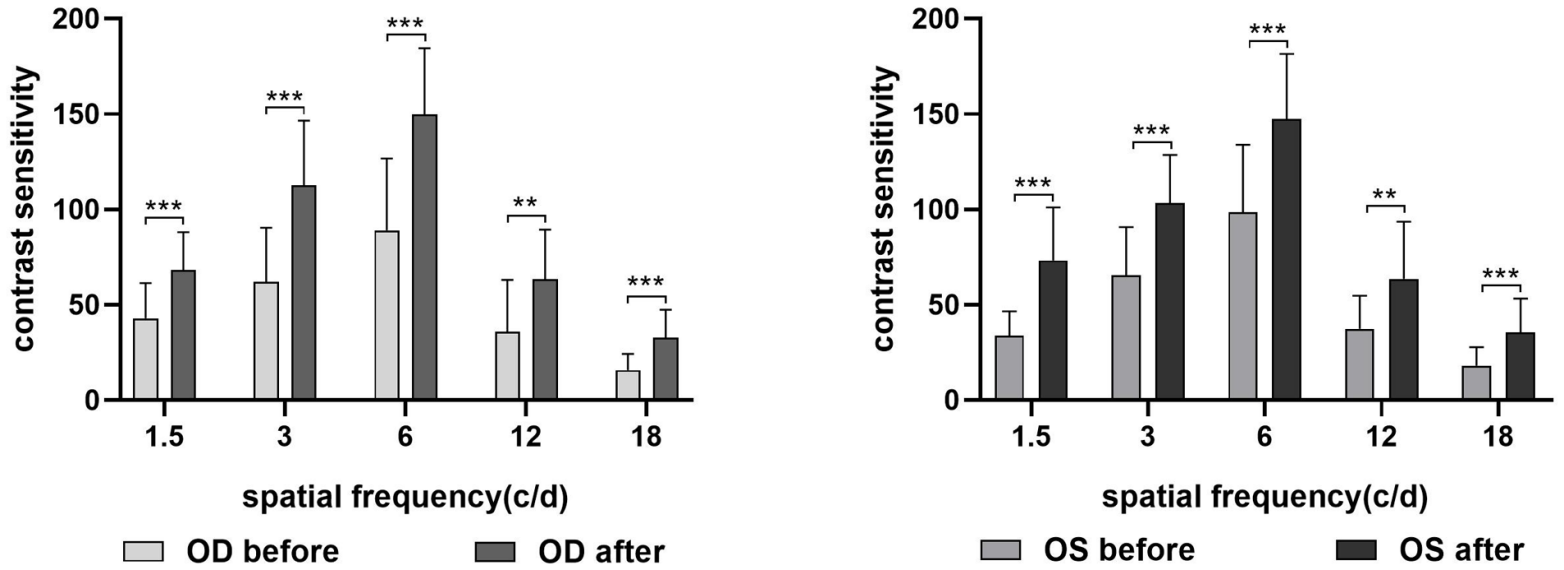
CS difference for each spatial frequency before and after acupuncture. OD, right eye; OS, left eye. **p_(FDR)_ < 0.01, ***p_(FDR)_ < 0.001.

Compared with before acupuncture, the CS value was increased at each spatial frequency (for the right eye: *p*_1.5cpd(FDR)_ = 0.000, *p*_3cpd(FDR)_ = 0.000, *p*_6cpd(FDR)_ = 0.000, *p*_12cpd(FDR)_ = 0.002, and *p*_18cpd(FDR)_ = 0.000; for the left eye: *p*_1.5cpd(FDR)_ = 0.000, *p*_3cpd(FDR)_ = 0.000, *p*_6cpd(FDR)_ = 0.000, *p*_12cpd(FDR)_ = 0.001, and *p*_18cpd(FDR)_ = 0.000) after acupuncture (Figure 3). The difference between the eyes was not statistically significant.

### 3.3 Microstates result

#### 3.3.1 Global explained variance

**Figure 4:**
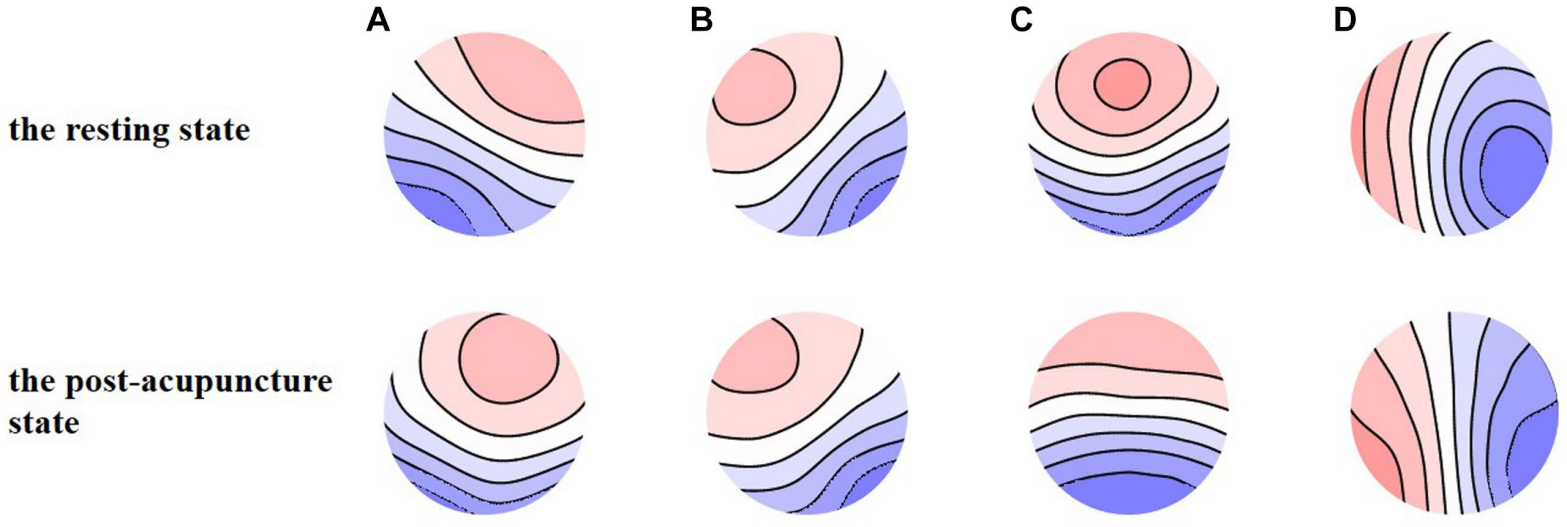
**(A-D)** EEG microstate maps during the resting state and the post-acupuncture state.

Figure 4 shows the four types of EEG microstate topography during the resting state and the post-acupuncture state, the global explained variance (GEV) of the resting state and the post-acupuncture state was 76.08 and 78.50%, separately. There was no statistical significance between them.

#### 3.3.2 Microstate parameters

**Figure 5:**
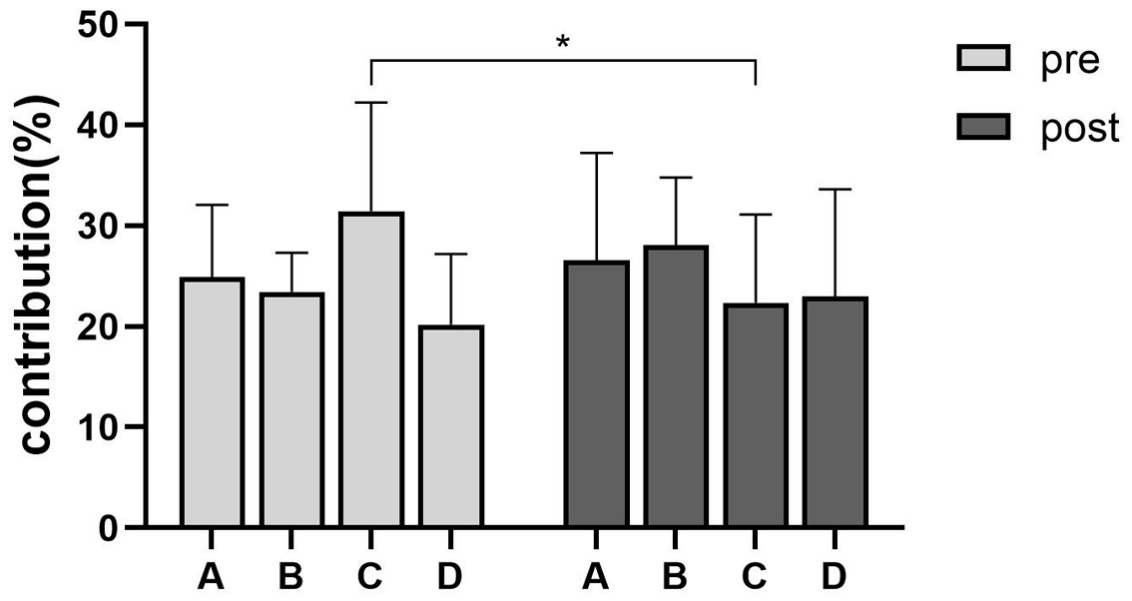
Contribution changes of microstate C during the resting state and the post-acupuncture state. *p_(FDR)_ < 0.05.

**Figure 6:**
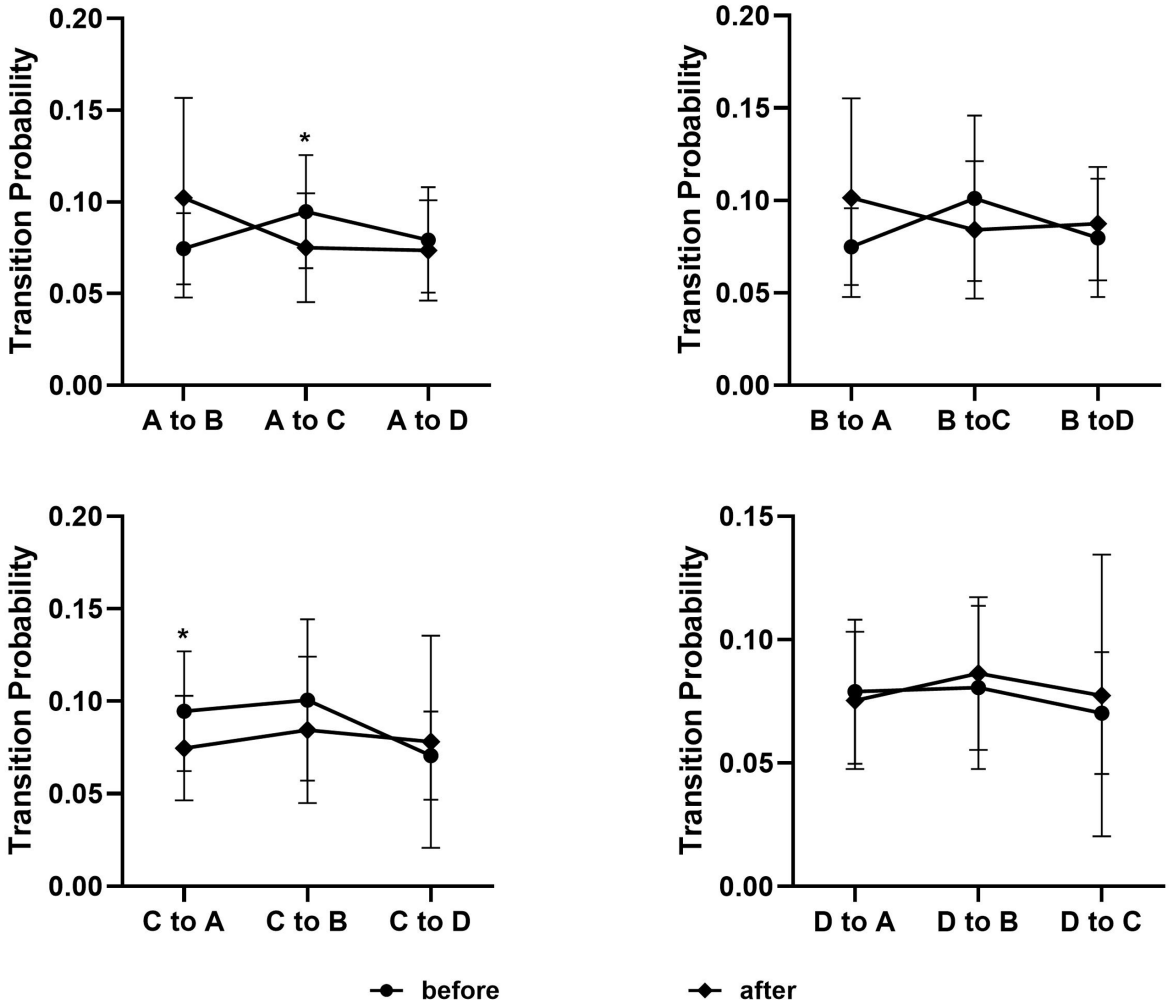
Transition probability between different microstates during the resting state and the post-acupuncture state. *p < 0.05.

The contribution of the microstate C showed a decrease during the post-acupuncture state compared with the resting state (*p*_(FDR)_ = 0.0325) (Figure 5). The duration and the occurrence had no statistical significance between the resting state and the post-acupuncture state. Compared with the resting state, the transition probability between microstate A and microstate C was decreased during the post-acupuncture state (*p*_A to C_= 0.019, *p*_C to A_= 0.022) (Figure 6).

#### 3.3.3 Correlation between microstate parameters and CS

**Figure 7:**
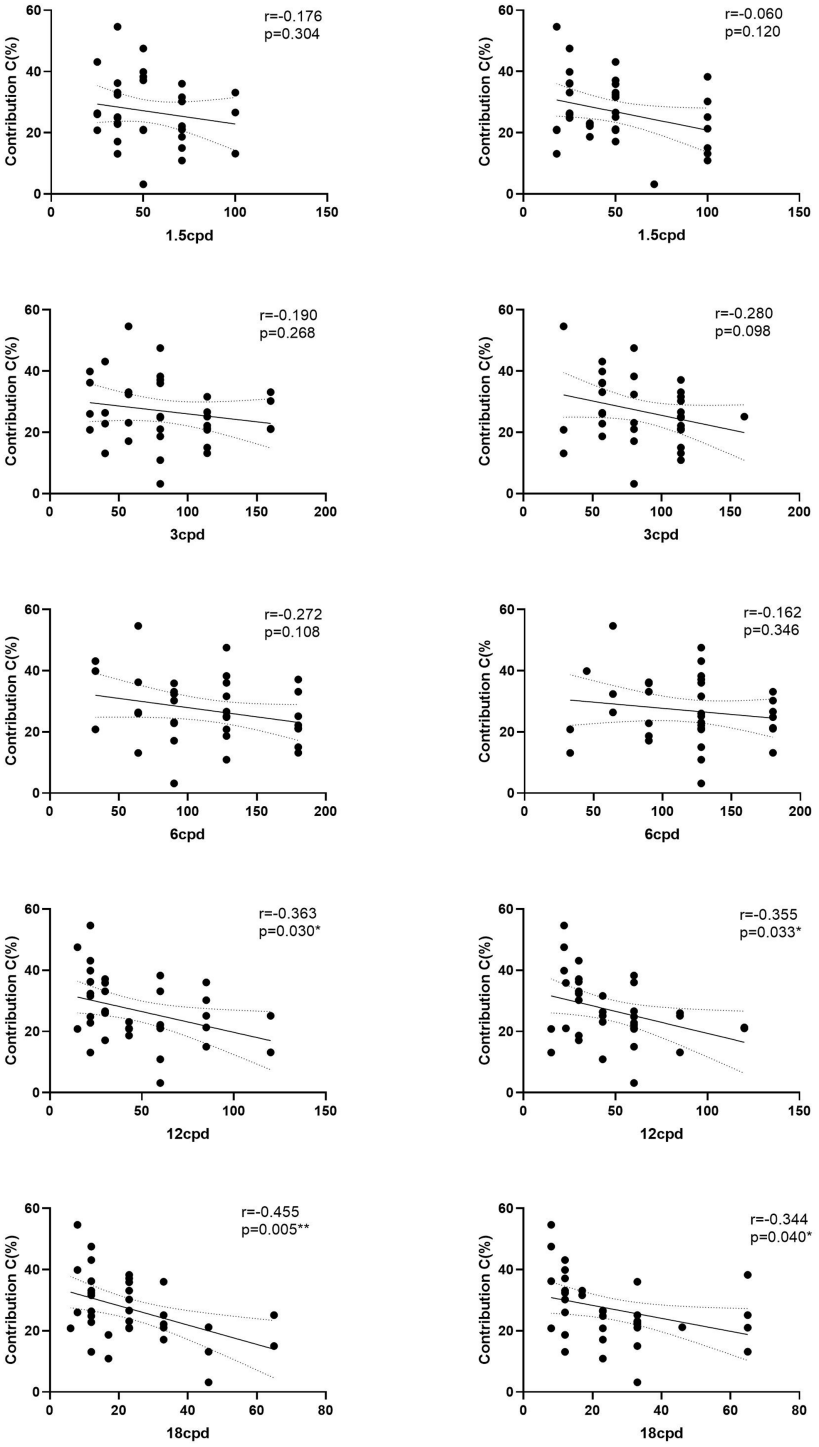
Correlation between contribution of microstate C and CS. The right panels are for the right eye, and the left panels are for the left eye. *p(FDR) < 0.05, **p(FDR) < 0.01.

Therefore, we performed a Pearson’s correlation analysis of the contribution and transition probability between microstate A and microstate C and the monocular CS. The contribution and CS of 12 and 18 cpd of the OD as right eye (RE) were negatively related (*r* = −0.363, *p* = 0.030; *r* = −0.455, *p* = 0.000); the results for the OS as left eye (LE) were consistent with those for the right eye (*r* = −0.355, *p* = 0.033; *r* = −0.344, *p* = 0.040). The correlation of the contribution and CS of left space frequencies had the same trend, but there was no statistical difference (Figure 7).

## 4 Discussion

In this study, we analyzed the effects of acupuncture on the right *Taiyang* acupoint of myopic patients on visual function and electroencephalographic activity to investigate the correlation between the improvement of visual function and the changes in the brain. The study found that acupuncture can improve the CS of patients with myopia, but it does not significantly improve the UDVA; acupuncture can reduce the contribution of microstate C and the transition probability between microstate A and microstate C. In addition, the contribution of microstate C was negatively correlated the monocular CS at 12 and 18cpd.

Although it has been shown that the tDCS of the posterior part of the head (occipital lobe) improves CS, this improvement is related to the pre-stimulation visual performance (the worse the pre-stimulation visual performance of a spatial frequency, the higher the gain of that spatial frequency after stimulation) (Wu et al., 2021). This study found that acupuncture at the right *Taiyang* acupoint of myopic patients increased CS at all spatial frequencies; this is different from the results of the above research. It may be due to the different mechanisms of acupuncture and the tDCS (Zhuang et al., 2013; Bello et al., 2023). In addition, research has found that the acupuncture at the anterior parts of the head can improve CS, which is consistent with the results of this study (Bittner et al., 2014; Blechschmidt et al., 2017).

According to fMRI-EEG multimodal studies, it has been shown that the neural components generating microstates overlapped with resting state networks independently identified with fMRI and that EEG-based microstates were strongly correlated with fMRI resting state networks (Zhao et al., 2022). Among them, microstate A corresponds to the auditory network, and microstate C is reflected in the salience network (Michel and Koenig, 2018; Ricci et al., 2022). Of these, the salience network, a high-order cognitive processing-related cortical network, can extract information from the most salient stimuli (Chen et al., 2016; Marstaller et al., 2021) and generate top-down feedback (Seeley et al., 2007), increasing sensitivity to relevant information (Lamichhane and Dhamala, 2015; Rijpma et al., 2022). It has been demonstrated that, compared with individuals in states of reduced attention (Brandeis and Lehmann, 1989), sleep (Brodbeck et al., 2012), hypnosis (Katayama et al., 2007), and with mental disorders (Giordano et al., 2018), normal individuals with higher sensitivity to environmental information have diminished microstate C (reduced contribution), reflecting a stronger connection to contextual information (Rieger et al., 2016). Therefore, the contribution of the microstate C during the post-acupuncture state decreases, which may reflect the closer connection of the individual and the background visual information, thereby improving the visual information processing capabilities. In addition, the transition probability between microstate A (auditory network) and microstate C was decreased during the post-acupuncture state, reflecting the reduction of salience network response to the auditory network. In this situation, the brain can focus more on visual information through the salience network, thereby promoting the extraction of relevant visual information. Therefore, the improvement of CS by acupuncture at the *Taiyang* acupoint may be achieved by increasing the efficiency of visual information extraction by salience network (Quentin et al., 2016).

This study found that, although the CS of all spatial frequencies after acupuncture increased, only CS at high spatial frequencies was negatively correlated with the contribution of microstate C. Compared with low space frequency (peripheral visual sensitivity), high space frequency (central fovea sensitivity) stimulation is more closely linked to high-level cognitive brain areas, such as the Fronto-Parietal network (Musel et al., 2013; Zhaoping, 2019; Sims et al., 2021). This showed that the contrast sensitivity of high spatial frequencies was negatively correlated with the contribution of microstate C, while the contrast sensitivity of low spatial frequencies showed a similar trend with these two parameters, but the correlation was not significant.

## 5 Limitations and future works

There were several potential limitations in this study. First, the sample size of this study was relatively small, and a large-scale study should be carried out to explore whether the brain mechanism of acupuncture to improve myopia CS is specific. Second, this study was conducted on myopic patients only. Future studies could incorporate emmetropia and compare the differences in brain dynamics between emmetropia and myopia after acupuncture, as well as the degree of improvement in CS between the two groups. In addition, this study only observed the CS of 20 min after acupuncture, and did not observe the degree of improvement for a longer period of time. In the future, a longitudinal follow-up study needs to be conducted to observe the length of time that CS improvement can be maintained by acupuncture.

## 6 Conclusion

In conclusion, the CS of both eyes was improved after acupuncture (20 min) at the *Taiyang* acupoint in myopic patients. At the same time, the contribution of microstate C decreased, which is negatively related to CS. This suggests that the contrast sensitivity of myopia was improved after acupuncture at the *Taiyang* acupoint, which may be related to the microstate C.

## Data availability statement

The research project has not yet been finalized and the data need to be further analysed.

## Ethics statement

The study protocol was reviewed and approved by the Ethics Committee of the Clinical Experimental Institution of the Eye Hospital Affiliated with Shandong University of Traditional Chinese Medicine (HEC-KS-2022019KY). The studies were conducted in accordance with the local legislation and institutional requirements. The participants provided their written informed consent to participate in this study.

## Author contributions

HB and JW conceptualized this study. HB supervised the study. KS, ZW, JM, and CZ collected data for this study. KS performed the statistical analyses. KS and LW drafted the manuscript. HB and JW were primarily responsible for the final content. All authors contributed to the article and approved the submitted version.
