# Corrigendum: The effect of acupuncture at the *Taiyang* acupoint on visual function and EEG microstates in myopia

**DOI:** 10.3389/fnint.2024.1367593

**Published:** 2024-03-15

**Authors:** Kangna Su, Lihan Wang, Zhongqing Wang, Jiayao Ma, Chao Zhang, Hongsheng Bi, Jianfeng Wu

**Affiliations:** ^1^Medical College of Optometry and Ophthalmology, Shandong University of Traditional Chinese Medicine, Jinan, China; ^2^Shandong Academy of Eye Disease Prevention and Therapy, Shandong Provincial Key Laboratory of Integrated Traditional Chinese and Western Medicine for Prevention and Therapy of Ocular Diseases, Shandong Provincial Clinical Research Center of Ophthalmology and Children Visual Impairment Prevention and Control, Shandong Engineering Technology Research Center of Visual Intelligence, Shandong Academy of Health and Myopia Prevention and Control of Children and Adolescents, Jinan, China; ^3^Ophthalmology Department of Northwest University First Hospital, Xi'an, Shaanxi, China; ^4^Affiliated Eye Hospital of Shandong University of Traditional Chinese Medicine, Jinan, China

**Keywords:** acupuncture, visual function, contrast sensitivity, microstates, EEG

In the published article, there was an error in the Funding statement. The grant number for the National Natural Science Foundation of China was incorrect in the original statement, which was: This work was supported by the National Natural Science Foundation of China (no. 8207151554), Shandong Medical and Health Science and Technology Development Plan Project (no. 202207020978), and Shandong Traditional Chinese Medicine Science and Technology Project (M-2023004). The correct Funding statement appears below.

